# Supplementary material for: Characterization of changes in the hemagglutinin that accompanied the emergence of H3N2/1968 pandemic influenza viruses
Source: PLoS Pathog. 2021 Sep 23;17(9):e1009566. doi: 10.1371/journal.ppat.1009566 (PMC8491938; doi:10.1371/journal.ppat.1009566)
Supplement: S1 Method — (DOCX) [file ppat.1009566.s001.docx]

**S1 Method. Construction of a GAMLSS model to analyse the frequency of observation of the HA mutants in the competitive replication assay**

In order to determine the effect of the inoculum titre on the frequency of each HK genotype, we employed a generalized linear model for location, scale and shape (GAMLSS model) under R environment [1]. GAMLSS models allow modelling not only the mean, but also location and scale parameters of the dependent variable distribution as linear parametric functions of explanatory variables [2].

The counts of each HA genotype were analyzed as number of “successes” of a beta binomial distribution in which the number of trials were given by the locus sequencing depth and the dispersion parameter allowed coping with the observed data overdispersion.

In GAMLSS models, the beta binomial distribution is depicted as BB(n, µ, σ), where n is the number of observations, µ is the event probability and is comprised in the range 0 < µ < 1 and σ is the dispersion parameter and is > 0. The mean E(Y) and variance Var(Y) are respectively expressed as nµ and $n\mu\left( 1-\mu\right)\left[ 1+\frac{\sigma}{1+\sigma}\left( n-1 \right) \right]$.

The probability density function of the beta binomial distribution as BB(n, µ, σ) is given by

| $p_{Y}\left( y\vert\mu, \right)= \frac{\left( n+ 1 \right)}{\left( y+1 \right)\left( n-y+1 \right)}\frac{\left( \frac{1}{\sigma} \right)\left( 1+\frac{\mu}{\sigma} \right)\left( n+\frac{\left( 1-\mu\right)}{\sigma}-y \right)}{\left( n+\frac{1}{\sigma} \right)\left( \frac{\mu}{\sigma} \right)\left( \frac{1-\mu}{\sigma} \right)}$ |  |
| --- | --- |
|  |  |

The independent variables considered in the model were the inoculum titre (expressed by the variable “dose”), the HA variant (expressed by the variable “SAP”) and the interaction of these two variables. The variable “dose” consisted of the levels “In”, “L”, “M”, and “H”, while “SAP” was described by the levels “HK-2”, “HK-62”, “HK-81”, “HK-92”, “HK-144” and “HK-193”. Internally to the model, the variable “dose” was coded by the dummy variables “doseL”, “doseM” and “doseH” which assumed values 1 when the “treatment” level was “L”, “M”, and “H” respectively and 0 otherwise. The “In” level was described by assigning the value 0 to all the “dose” dummy variables. Similarly, the variable “SAP” was expressed by the dummy variables “HK2”, “HK62”, “HK81”, “HK92”, “HK144” and “HK193” which assumed value 1 when the described SAP was HK-2, HK-62, HK-81, HK-92 and HK-193 respectively, and 0 otherwise. The HK-144 genotype was described by assigning the value 0 to all the “SAP” dummy variables.

The final model consisted of 2 parts, the one modelling the frequency $\mu$

$$\log\left( \frac{\mu}{1-\mu} \right)={Intercept}_{\mu}+ \hat{\beta}_{\mu\_doseH} doseH+ \hat{\beta}_{\mu\_doseM} doseM+ \hat{\beta}_{\mu\_doseL} doseL+\hat{\beta}_{\mu\_HK2} HK2+ \hat{\beta}_{\mu\_HK62} HK62+ \hat{\beta}_{\mu\_HK81} HK81+ \hat{\beta}_{\mu\_HK92} HK92+\hat{\beta}_{\mu\_HK193} HK193+\hat{\beta}_{\mu\_doseH\_HK2} doseH HK2+\hat{\beta}_{\mu\_doseM\_HK2} doseM HK2+\hat{\beta}_{\mu\_doseL\_HK2} doseL HK2+ \hat{\beta}_{\mu\_doseH\_HK62} doseH HK62+ \hat{\beta}_{\mu\_doseM\_HK62} doseM HK62+ \hat{\beta}_{\mu\_doseL\_HK62} doseL HK62+ \hat{\beta}_{\mu\_doseH\_HK81} doseH HK81+ \hat{\beta}_{\mu\_doseM\_HK81} doseM HK81+ \hat{\beta}_{\mu\_doseL\_HK81} doseL HK81+ \hat{\beta}_{\mu\_doseH\_HK92} doseH HK92+ \hat{\beta}_{\mu\_doseM\_HK92} doseM HK92+ \hat{\beta}_{\mu\_doseL\_HK92} doseL HK92+ \hat{\beta}_{\mu\_doseH\_HK193} doseH HK193+ \hat{\beta}_{\mu\_doseM\_HK193} doseM HK193+ \hat{\beta}_{\mu\_doseL\_HK193} doseL HK193$$

and the one modelling the dispersion parameter

$$\log\left( \sigma\right)={Intercept}_{\sigma}+ \hat{\beta}_{\sigma\_doseH} doseH+ \hat{\beta}_{\sigma\_doseM} doseM+ \hat{\beta}_{\sigma\_doseL} doseL$$

The model parameter estimates are given in the Table A. The significance of each predictive variable for µ and σ was assessed via likelihood ratio test (Table B).

**Table A.** Parameter estimates (Est) and 95% confidence intervals (95% CI) of the GAMLSS model for the analysis of the frequency of observation of the HA mutants in the competitive replication assay.

| **Frequency modelling** | |  |  |
| --- | --- | --- | --- |
| *Predictors* | *Est* | | *95% CI* |
| $\mathrm{Intercept}_{\mu}$ | -0.56 | | (-0.69, -0.43) |
| $\hat{\beta}_{\mu\_doseH}$ | -0.27 | | (-0.53, -0.01) |
| $\hat{\beta}_{\mu\_doseM}$ | -0.35 | | (-1.23, 0.52) |
| $\hat{\beta}_{\mu\_doseL}$ | -0.99 | | (-2.16, 0.19) |
| $\hat{\beta}_{\mu\_HK2}$ | -2.07 | | (-2.37, -1.78) |
| $\hat{\beta}_{\mu\_HK162}$ | -1.18 | | (-1.41, -0.96) |
| $\hat{\beta}_{\mu\_HK81}$ | -0.99 | | (1.21, -0.78) |
| $\hat{\beta}_{\mu\_HK92}$ | -1.40 | | (-1.63, -1.16) |
| $\hat{\beta}_{\mu\_HK193}$ | -1.60 | | (-1.85, -1.35) |
| $\hat{\beta}_{\mu\_doseH\_HK2}$ | 0.91 | | (0.43, 1.40) |
| $\hat{\beta}_{\mu\_doseM\_HK2}$ | 1.62 | | (0.37, 2.88) |
| $\hat{\beta}_{\mu\_doseL\_HK2}$ | 2.36 | | (0.86, 3.86) |
| $\hat{\beta}_{\mu\_doseH\_HK62}$ | 0.11 | | (-0.33, 0.54) |
| $\hat{\beta}_{\mu\_doseM\_HK62}$ | -0.47 | | (-1.90, 0.95) |
| $\hat{\beta}_{\mu\_doseL\_HK62}$ | -0.50 | | (-2.41, 1.42) |
| $\hat{\beta}_{\mu\_doseH\_HK81}$ | -0.18 | | (-0.61, 0.26) |
| $\hat{\beta}_{\mu\_doseM\_HK81}$ | -0.21 | | (-1.53, 1.12) |
| $\hat{\beta}_{\mu\_doseL\_HK81}$ | -0.81 | | (2.72, 1.11) |
| $\hat{\beta}_{\mu\_doseH\_HK92}$ | 0.18 | | (-0.28, 0.63) |
| $\hat{\beta}_{\mu\_doseM\_HK92}$ | -0.01 | | (-1.38, 1.37) |
| $\hat{\beta}_{\mu\_doseL\_HK92}$ | -0.06 | | (-1.86, 1.73) |
| $\hat{\beta}_{\mu\_doseH\_HK193}$ | 0.25 | | (-0.22, 0.72) |
| $\hat{\beta}_{\mu\_doseM\_HK193}$ | -0.09 | | (-1.48, 1.29) |
| $\hat{\beta}_{\mu\_doseL\_HK193}$ | 1.31 | | (-0.25, 2.88) |
|  |  | |  |
| **Sigma modelling** |  | |  |
| *Predictors* | *Est* | | *95% CI* |
| $\mathrm{Intercept}$ | -7.10 | | (-7.19, -7.02) |
| $\hat{\beta}_{\sigma\_doseH}$ | 2.11 | | (1.94, 2.27) |
| $\hat{\beta}_{\sigma\_doseM}$ | 5.89 | | (5.32, 6.45) |
| $\hat{\beta}_{\sigma\_doseL}$ | 6.75 | | (5.99, 7.51) |

**Table B.** Analysis of deviance table (type II likelihood ratio tests) of the GAMLSS model for the analysis of the frequency of observation of the HA mutants in the competitive replication assay. The model relates the frequency to the variables “dose”, “SAP” and their interaction, and the dispersion parameter sigma to “dose” alone.

| **Frequency modelling** |  |  |  |  |
| --- | --- | --- | --- | --- |
| *Predictors* | *npar* | *AIC* | *Χ ^2^* | *p* |
| <none> |  | 2617.1 |  |  |
| dose:SAP | 15 | 2650.2 | 63.17 | 7.14x10^-8^ |
| **Sigma modelling** |  |  |  |  |
| *Predictors* | *npar* | *AIC* | *Χ ^2^* | *p* |
| dose | 3 | 2786.8 | 175.79 | 7.15x10^-38^ |

Legend: *npar =* number of parameters associated to the relative predictor; *AIC =* Akaike’s information criterion value observed upon dropping of the relative predictor; *Χ ^2^ =* likelihood ratio test statistic; p = likelihood ratio test *p* value.

**Construction of a GAMLSS model for analysis proportions of HK parent genotype**

Whereas the 6 HK mutants were unambiguously identified in the mixtures via unique SNPs, the fraction of the parent HK virus could only be inferred by subtraction of the mutants’ proportions from a theoretical value of 1. In order to tentatively compare the mean values of the proportions of the different mutants with the ones of the parent strain, we calculated the hypothetical number of reads referable to HK by multiplying the inferred HK proportions with the mode of the sequencing depths of the analyzed loci (3300). Similarly to the other mutants, the parent HK means were derived from a beta binomial model considering the observed mutant counts and the ones hypothesised for the parent genotype (data not shown). This last model served only as a tool to roughly estimate the mean of the parent HK genotype proportions and was not meant for precise parameter estimation or significance analysis.

**References**

1. R Core Team. R: A Language and Environment for Statistical Computing. R Foundation for Statistical Computing, Vienna, Austria, 2018. Available from <https://www.r-project.org/>

2. Rigby RA, Stasinopoulos DM. Generalized additive models for location, scale and shape. J R Stat Soc Ser C Appl Stat. 2005;54:507–554. doi: 10.1111/j.1467-9876.2005.00510.x.
