# Supplementary material for: Characterization of changes in the hemagglutinin that accompanied the emergence of H3N2/1968 pandemic influenza viruses
Source: PLoS Pathog. 2021 Sep 23;17(9):e1009566. doi: 10.1371/journal.ppat.1009566 (PMC8491938; doi:10.1371/journal.ppat.1009566)
Supplement: S3 Table — (PDF) [file ppat.1009566.s005.pdf]

S3 Table. P values for the differences between Kass of viral complexes with SGPs presented in the Fig 3a. Values lower than 0.05 are highlighted with yellow.

SGPs compared	Virus			
	**R7**	**R2**	**mal-H3N8**	**rt-H7N7**
3'SLN - $SiaLe^c$	1.0000	1.0000	0.3075	0.6782
3'Sia-TF - $SiaLe^c$	0.5529	0.5431	0.0129	0.0608
6-Su-3'SLN - $SiaLe^c$	1.0000	1.0000	0.5209	0.0002
$SiaLe^x$ - $SiaLe^c$	0.0000	0.0204	0.0000	0.0000
6-Su-$SiaLe^x$ - $SiaLe^x$	0.0000	0.0028	0.0011	0.0000
3'Sia-TF - 3'SLN	0.1037	0.5431	0.0000	0.6782
6-Su-3'SLN - 3'SLN	1.0000	1.0000	0.0703	0.0207
$SiaLe^x$ - 3'SLN	0.0000	0.0217	0.0071	0.0000
6-Su-$SiaLe^x$ - 3'SLN	0.0000	0.0030	0.1301	0.0002
6-Su-3'SLN - 3'Sia-TF	0.5529	1.0000	0.1301	0.5230
$SiaLe^x$ - 3'Sia-TF	0.0000	0.0000	0.0000	0.0071
6-Su-$SiaLe^x$ - 3'Sia-TF	0.0000	0.0000	0.0000	0.0254
$SiaLe^x$ - 6-Su-3'SLN	0.0000	0.0030	0.0000	0.5230
6-Su-$SiaLe^x$ - 6-Su-3'SLN	0.0000	0.0003	0.0000	0.6782
6-Su-$SiaLe^x$ - $SiaLe^x$	1.0000	1.0000	0.5209	0.6782
