# Supplementary figures and images for: Characterization of changes in the hemagglutinin that accompanied the emergence of H3N2/1968 pandemic influenza viruses
Source: PLoS Pathog. 2021 Sep 23;17(9):e1009566. doi: 10.1371/journal.ppat.1009566 (PMC8491938; doi:10.1371/journal.ppat.1009566)

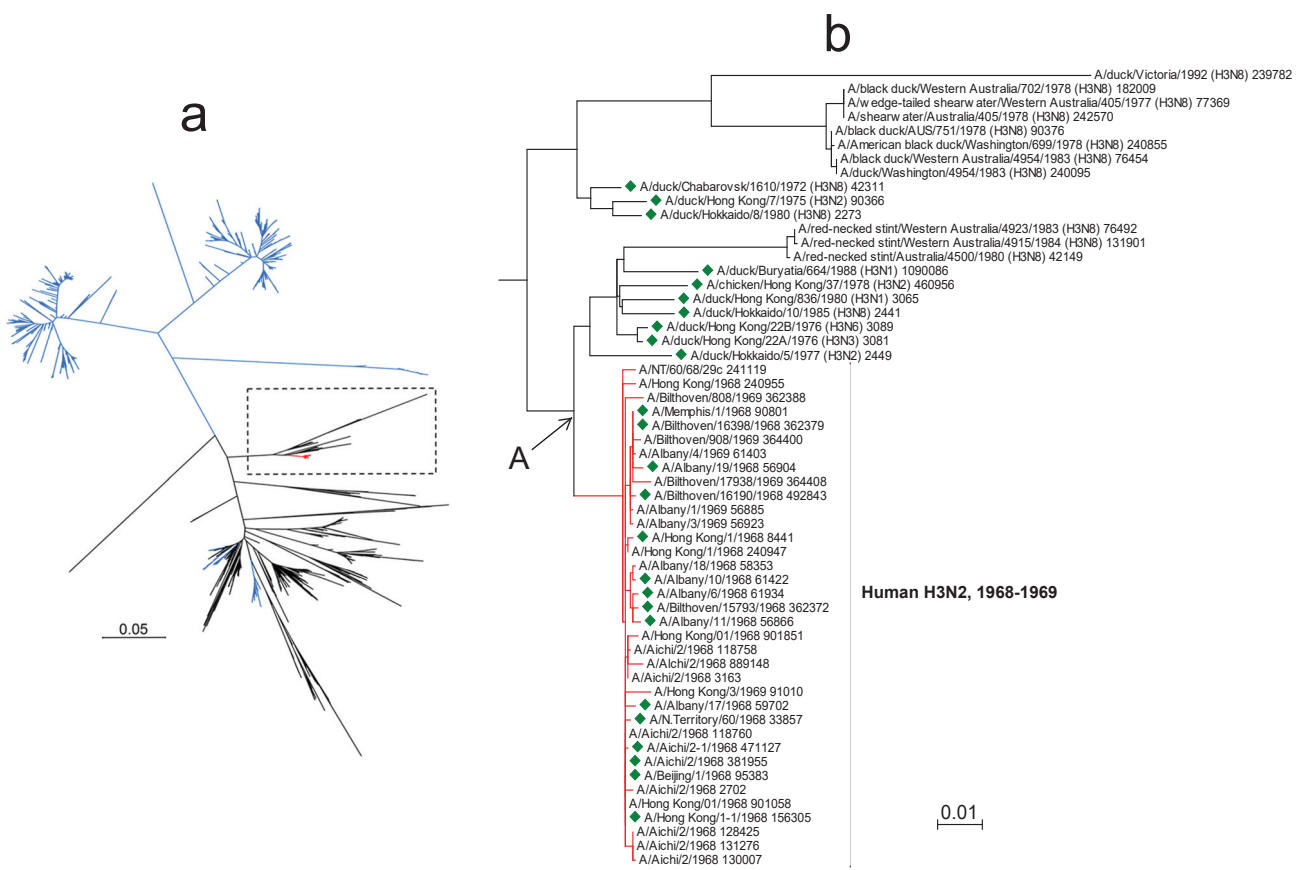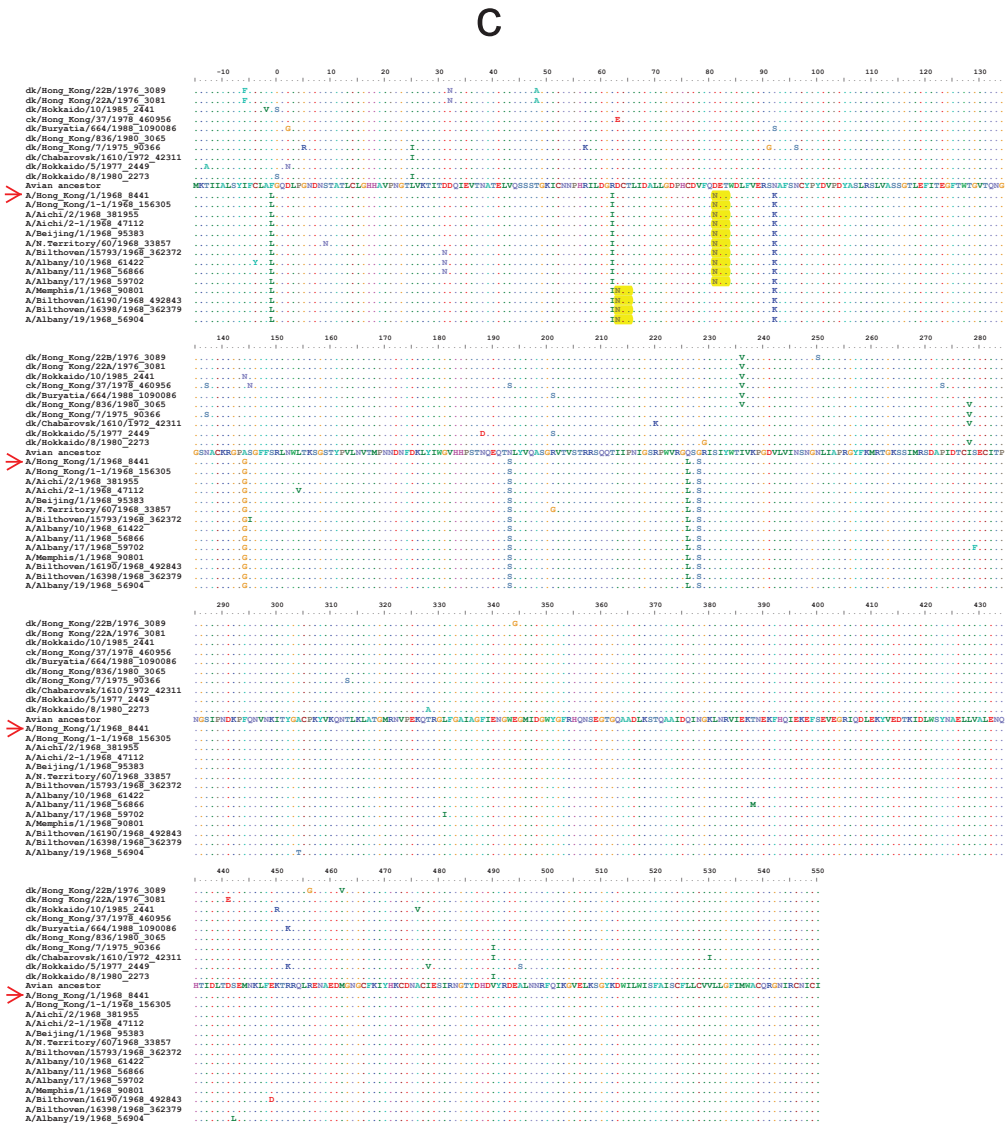

Supplement: S1 Fig — (a) Phylogenetic relationships between H3N2/1968 and avian IAVs. All full-length HA sequences of avian H3 IAVs and of pandemic IAVs isolated in 1968–1969 were downloaded from the GISAID EpiFlu database and processed as described in Materials and Methods. The final dataset contained sequences of 1494 avian and 36 human IAV HAs. The evolutionary history was inferred using MEGA7 with the maximum likelihood method based on the Kimura 2-parameter model. The tree is drawn to scale, with branch lengths measured in the number of nucleotide substitutions per site. Colours depict avian IAVs from North America (blue), Eurasia and Oceania (black), and H3N2/1968 pandemic viruses (red). (b) Detailed view of the branch, which includes pandemic and the closest avian IAVs marked by the dashed box in the panel S1a. Subtypes of avian IAVs and accession numbers of all sequences are shown next to the strain names. HA amino acid sequence of the common avian-human ancestor (node A) was inferred using the Ancestors program of MEGA7. The ancestral sequence together with the sequences of representative avian and human IAVs depicted by green diamonds are shown in the panel S1c. (c) HA amino acid sequences of H3N2 pandemic IAVs viruses isolated in 1968, the closest avian IAVs and the inferred common avian-human ancestor. Numbering starts from the N-terminus of mature HA protein, the signal peptide is numbered from -15 to 0. Dots depict identity with the sequence of the avian ancestor. Glycosylation sites at HA positions 63–65 and 81–83 are highlighted by yellow. Arrow depicts virus strain A/Hong Kong/1/1968 (H3N2) used to make recombinant viruses in this study. The figure was generated using Bio-Edit. (PDF) [file ppat.1009566.s006.pdf]

1975-2019

a

b

Avian ancestor

0.005

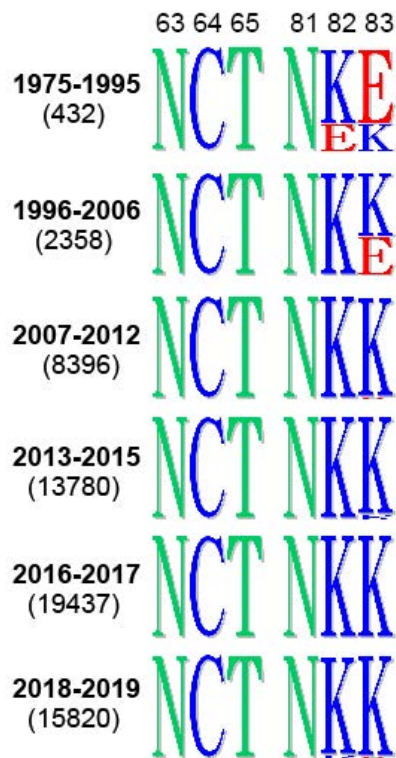

Supplement: S2 Fig — (a) Phylogenetic relationships between the HA of IAVs isolated from 1968 to 1995 were inferred using MEGA7 with the maximum likelihood method. GISAID EpiFlu accession numbers and amino acids in positions 63–65 and 81–83 are shown next to the strain names. Coloured circles depict presence of glycosylation sites 63–65 (blue) and 81–83 (red). One virus strain (A/Port Chalmers/1/1973) contained glycosylation sites at both positions (magenta). The branch containing sequences of the viruses isolated after 1975 is collapsed for clarity (blue triangle). Prototype strains used to define two glycosylation lineages of pandemic viruses are highlighted by yellow. (b) Protein logos show frequencies of amino acids at positions 63–65 and 81–83 of the HA. Years of virus isolation and numbers of analysed sequences (in parentheses) are shown on the left. The figure was generated using Phylo-mLogo software (Shih ACC, Lee DT, Peng CL, Wu YW. Phylo-mLogo: an interactive and hierarchical multiple-logo visualization tool for alignment of many sequences. BMC bioinformatics. 2007; 8: 63). (PDF) [file ppat.1009566.s007.pdf]

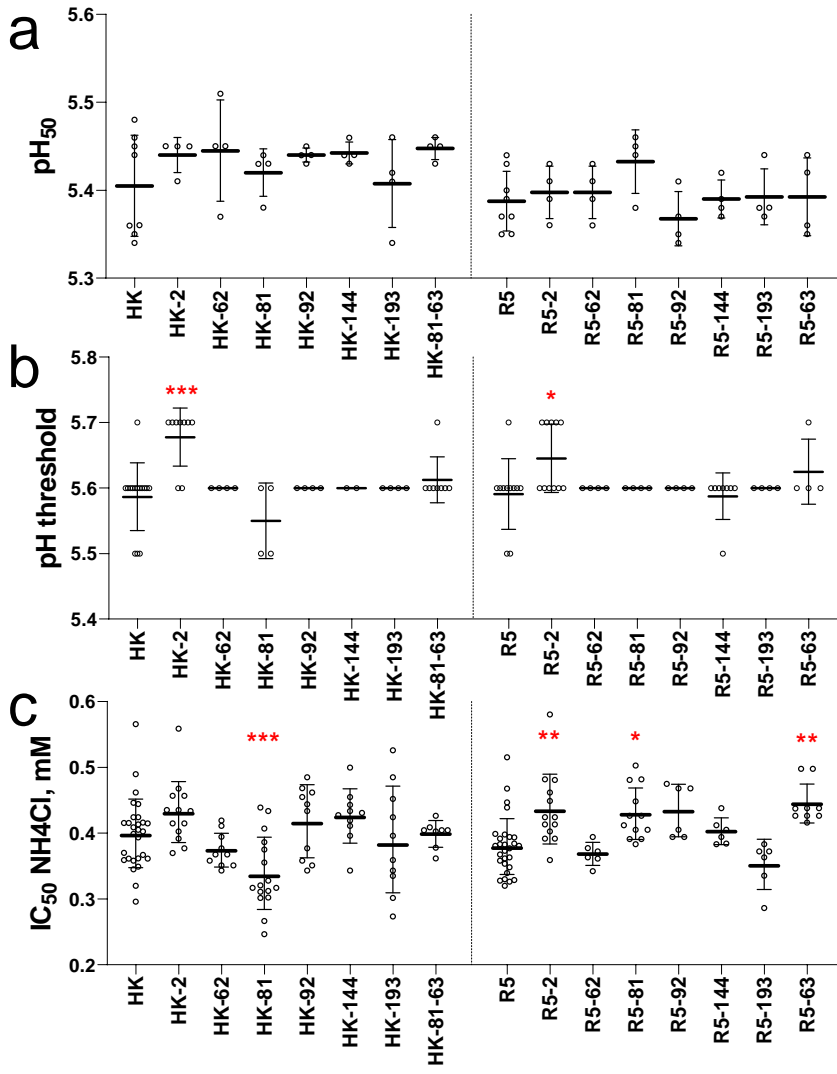

Supplement: S3 Fig — pH of acid-induced conformational transition (a), pH threshold of polykarion formation (b), and inhibition of viral infection by ammonium chloride (c) were assayed as described in Materials and Methods. Panels show data points, geometric mean and SDs from 2 to 8 experiments performed on different days with 2 to 6 replicates each. Data in panels S3a and S3c were analysed using general linear mixed models in R 3.6.0. Concentration was log-transformed before analysis. Day was included as a random intercept. Figures show data adjusted for day. In panel S3b, due to the low resolution of the assay (with only 3 distinct pH values), pH was analysed as an ordinal variable using an ordered logistic regression model. Multiple tests of contrasts within these models were done using simultaneous tests for general linear hypotheses, and P values were adjusted using the single step method. In all panels, vertical dotted line separates point mutants of HK and point mutants of R5. Red asterisks depict point mutants that were significantly different from their parental viruses, either HK or R5. No significant difference was observed between HK and R5 in these assays. (PDF) [file ppat.1009566.s008.pdf]

a

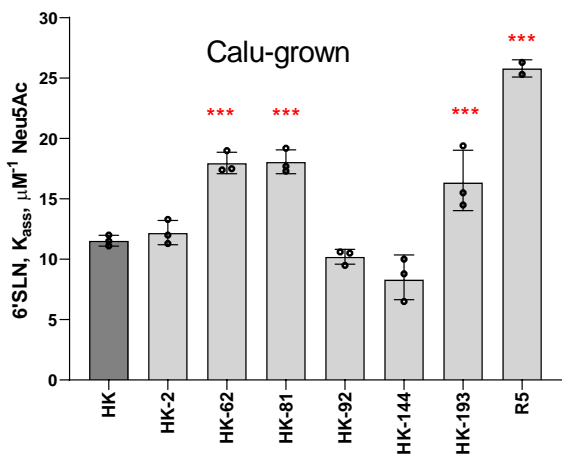

b

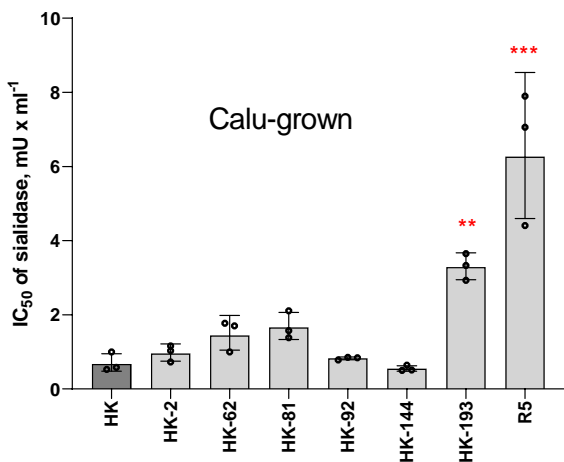

c

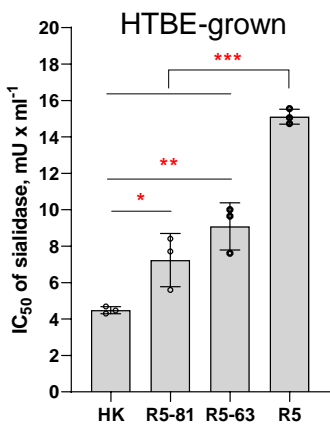

Supplement: S4 Fig — (a) Association constants of viral complexes with 6’SLN (20 kDa). (b,c) Virus avidity for receptors on MDCK cells expressed as concentrations of the Vibrio cholerae sialidase that reduced numbers of infected cells by 50% (IC50). The higher IC50, the higher binding avidity. All panels show replicates, mean values (bars) and SDs. Asterisks in panels S4a and S4b show P values for the differences with HK (dark grey bars) determined by one-way Anova with Dunnett’s multiple comparisons test. Asterisks in panel S4c depict differences between individual viruses determined by one-way Anova with Tukey’s test. (PDF) [file ppat.1009566.s009.pdf]

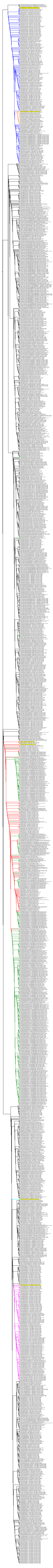

Supplement: S5 Fig — The figure shows maximum likelihood tree for the nucleotide sequences of H3 HA inferred using IQ-TREE 2 and plotted using Mega 7. Taxon labels (for example, A/equine/Miami/1/1963-A/H3N8-129722-H-R-NCT-YEN-S-A-K-QSG) include virus name, subtype, GISAID EpiFlu accession number and amino acids in HA positions -2, 62, 63–65, 81–83, 92, 144, 193, 226–228. Colours depict the following stable host-specific lineages. Black, avian; red, human; green, swine; blue, equine; cyan, equine/Jilin/1/1989; orange, canine H3N8; magenta, canine H3N2. The earliest virus isolates from mammalian lineages are highlighted with yellow. Green dots depict 10 sporadic avian-like mammalian isolates. The tree includes the following full-length non-redundant sequences available from GISAID EpiFlu database: 106 representative sequences of human IAVs isolated from 1968 to 2020 (2 sequences per year); 456 representative sequences of swine IAVs selected from the total 2663 sequences using JalView; all 402 sequences of other mammalian IAVs (mainly equine and canine); all 1494 sequences of avian IAVs; 31 sequences of avian, swine and human IAVs sporadically isolated from a heterologous host species. (PDF) [file ppat.1009566.s010.pdf]

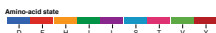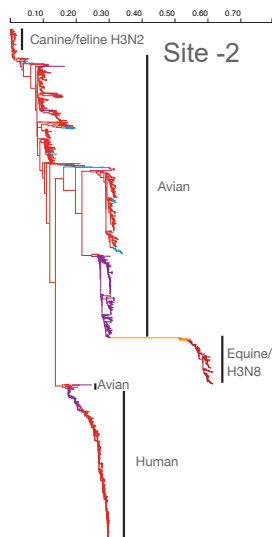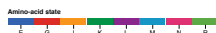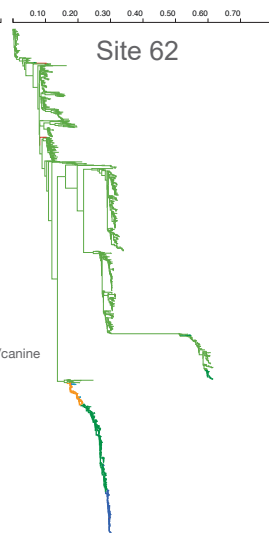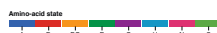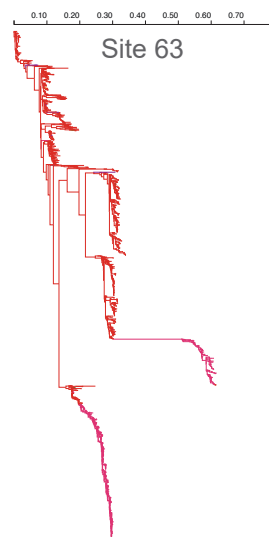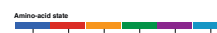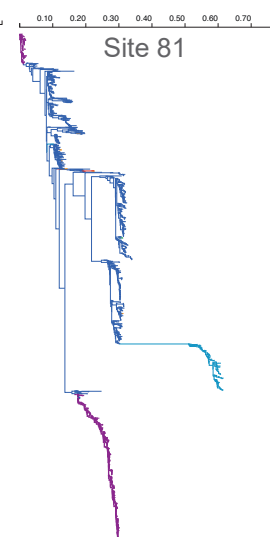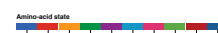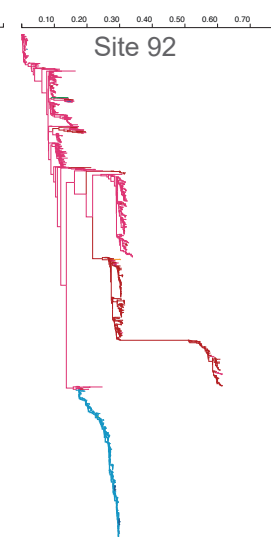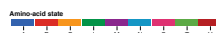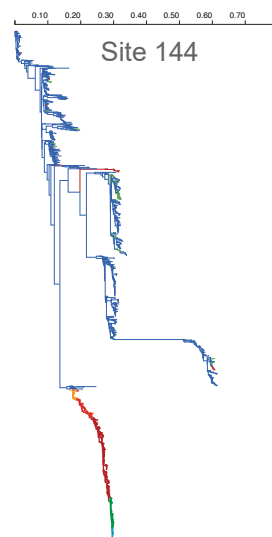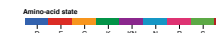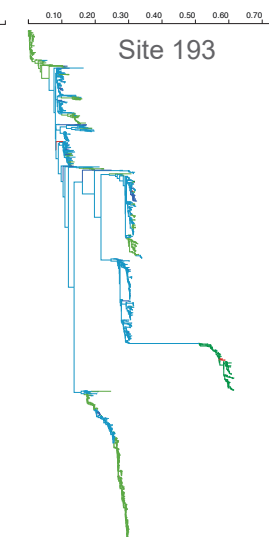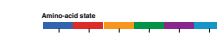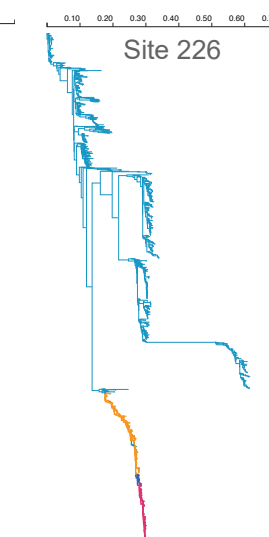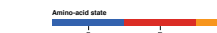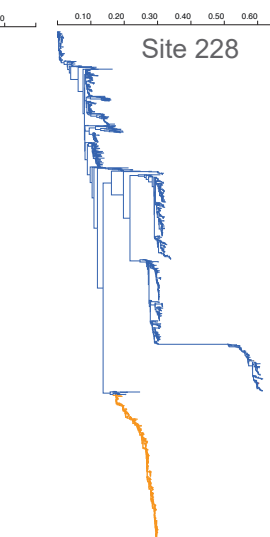

Supplement: S6 Fig — The tree is based on HA sequences used for selection pressure analyses and includes 1492 sequences of avian IAVs, 406 sequences of equine, canine, feline and seal IAVs and 803 sequences of human IAVs isolated in the years from 1968 to 1999. Unobserved ancestral codons were inferred using the SLAC method. Host-specific clades are depicted in the tree display for the site -2. (PDF) [file ppat.1009566.s011.pdf]
